# mTOR Signaling Network in Cell Biology and Human Disease

**DOI:** 10.3390/ijms232416142

**Published:** 2022-12-18

**Authors:** Jane J. Yu, Elena A. Goncharova

**Affiliations:** 1 Department of Internal Medicine, College of Medicine, University of Cincinnati, Cincinnati, OH 45267, USA; 2 Division of Pulmonary, Critical Care and Sleep Medicine, Department of Internal Medicine, Lung Center, School of Medicine, University of California Davis, Davis, CA 95616, USA

The mechanistic target of rapamycin (mTOR) is a serine/threonine protein kinase that regulates multiple processes, including gene transcription, protein synthesis, ribosome biogenesis, autophagy, cell metabolism, and cell growth. mTOR acts as a catalytic core for two functionally distinct complexes, mTOR complex 1 (mTORC1), which contains RAPTOR and phosphorylates p70 S6 kinase (S6K) and the translational repressor 4E-binding protein 1 (4E-BP1) to regulate protein synthesis, and mTORC2, containing RICTOR, which phosphorylates serin-threonine protein kinase AKT at Ser473 to control cytoskeletal organization, cell motility, and cell survival [1]. Because of its fundamental function as a modulator of cell behavior in response to multiple environmental stimuli, mTOR plays an important role in normal human physiology and various human diseases, including pulmonary lymphangioleiomyomatosis (LAM), the tuberous sclerosis complex (TSC), cancer, cardiovascular and inflammatory diseases, metabolic syndrome, and aging.

The dysregulation of mTOR kinase activity can be caused by various factors, including genetic alterations, epigenetic components, an imbalance of upstream mTOR regulators, and post-translational modifications (PTMs). The activation of oncogenes and inactivation of tumor suppressors contribute to the dysregulation of the mTOR signaling network, causing aberrant cell growth (increase in size) and proliferation (increase in cell number). These cell autonomous processes are also regulated by growth factors and mitogens secreted by neighboring cells. The recent review article by Popova and Jücker [2] provides a comprehensive overview of the phosphatidylinositol 3-kinase (PI3K)/AKT/mTORC1 signaling pathway, as well as its role, mechanisms of regulation and function in cancer. This review also presents up-to-date data regarding PI3K/AKT/mTOR inhibitors in clinical studies, or those currently approved for the treatment of human cancers, and discusses the potential problems of drug resistance and adverse effects [2]. Further expanding our knowledge of mTOR regulation and function, the review by Yin et al. [3] summarizes current knowledge on the PTM-mediated regulation of mTORC1 and mTORC2 networks and discusses recent progress in targeting the mTOR pathway and PTM-related enzymes to treat human diseases [3], reinforcing the translational importance of studying PTMs of the mTOR network in health and disease.

Several mTORC1 inhibitors (rapamycin and rapalogs) have been approved for the treatment of various diseases, including cancers, but the search for clinically attractive, potent, and selective mTORC2 inhibitors is currently ongoing. The study by Guenzle et al. evaluated the efficacy of the mTORC2-specific inhibitor JR-AB2-011 in liver metastasis from melanoma, which is still associated with poor prognosis [4]. This study showed that JR-AB2-011 treatment significantly reduced the migration and invasion capacity of melanoma cells by impairing the activation of matrix metalloproteinase-2 (MMP2), an important player in angiogenesis and cancer [5]. Further reinforcing the preclinical importance of this study, the authors demonstrated that this mTORC2 inhibitor induced cell death by non-apoptotic pathways and reduced tumor cell proliferation in a dose-dependent manner. Furthermore, using in vivo imaging and necropsy, the authors demonstrated that JR-AB2-011 significantly reduced the liver metastasis of melanoma cells in a syngeneic murine metastasis model [4]. This study highlights the potential of the pharmacological inhibition of mTORC2 as a potent novel anti-cancer approach to liver metastasis from melanoma in preclinical settings, providing a novel potential avenue for therapeutic intervention.

Cancer patients often face substantial challenges to bone health, the maintenance of which is problematic due to various complications, including bone metastasis and the side effects of anti-cancer treatments. Interestingly, the mTORC1 inhibitor everolimus showed significant clinical benefits in breast cancer patients by reducing the negative effects of estrogen suppression on bone health [6]. mTOR is also shown to be important to normal bone homeostasis, and both the positive and negative consequences of mTORC1 inhibition on bone health have been reported [7], calling for a further investigation of the mechanisms of mTOR action. A recent study by Rybchyn et al. [8] uncovered a novel mechanism of Homer1/Calcium-sensing receptor (CaSR)-dependent activation of mTORC2-Akt, which, in turn, induced stabilization of β-catenin and activation of mTORC1 in osteoblasts. In their further study [9], the authors identified novel roles for Homer1 as a regulator of CaSR protein levels and subcellular localization in cells of the osteoblast lineage [8], emphasizing the complexity of the regulation of mTOR signaling.

The mutational inactivation of the growth suppressors tuberous sclerosis complex 1 (TSC1) and TSC2, upstream inhibitors of mTORC1, is linked with pulmonary LAM and TSC syndrome. LAM is a slowly progressive, low-grade, metastasizing neoplasm in women, characterized by the infiltration of the lung parenchyma with abnormal smooth muscle-like cells, resulting in cystic lung destruction [10]. The invading cells in LAM arise from an unknown source and harbor mutations in TSC1 or TSC2 genes, which encode TSC1 and TSC2 proteins. TSC1 and TSC2 form a heterodimeric GTP-ase activating protein (GAP) with activity toward the Ras homolog enriched in the brain (Rheb), an activator of mTORC1. Thus, the loss of either TSC1 or TSC2 results in the constitutive activation of mTOR [11], dysregulated cellular proliferation, and a program of frustrated lymphangiogenesis, culminating in disordered lung remodeling and respiratory failure. A study by Huang et al. reported the first histological structural analysis in LAM patients with and without treatment with mTORC1 inhibitor rapamycin (sirolimus) [12]. Sirolimus treatment decreased the immunoreactivity of mTOR and human melanoma black 45 (HMB45) and reduced the phosphorylation of cofilin, an actin-binding protein, in LAM lung lesions. Sirolimus also reduced interstitial septal thickness, indicating a novel mechanism by which sirolimus protects against cystic destruction in patients with LAM. Moreover, primary cells were isolated and developed from the LAM lung tissues for functional analyses, including cell migration and proliferation in response to sirolimus treatment. This study provided novel evidence that sirolimus effectively suppresses the mTOR/cofilin pathway to inhibit the migration and proliferation of LAM cells.

TSC is an autosomal dominant disorder with multi-system manifestations, including the development of benign neoplasms in the brain, heart, kidneys, and lungs [13]. Complications associated with TSC tumors include cognitive impairment, intractable seizures, autism, renal hemorrhage and insufficiency, and respiratory failure. TSC is caused by germline inactivating mutations in TSC1 and TSC2 and consecutive mTOR up-regulation. Not surprisingly, mTORC1 inhibitors have been used for the treatment of TSC manifestations [13]. Although mTORC1 inhibitors often shrink tumors and improve clinical outcomes, the results from multiple clinical trials indicate that mTORC1 inhibitors do not induce remission in TSC and there are risks associated with the long-term use of sirolimus.

To identify novel molecular target pathways responsible for TSC and LAM pathogenesis and progression, cutting-edge single-cell RNA sequencing (scRNA-seq) studies have been performed and have identified a unique population of cells expressing LAM signature genes [14,15]. Among the numerous signaling pathways that are activated in unique LAM cells, Evans et al. reported the crosstalk between mTORC1 and Wnt/β-catenin pathways [14]. Their original research studies identified the aberrant up-regulation of the Wnt signaling pathway that leads to alveolar enlargement, one of the main characteristics of pulmonary LAM. They also found that rapamycin treatment slowed down the progression of this phenotype, and importantly, the genetic ablation of the β-catenin gene *cttnb1* ameliorated alveolar enlargement in a novel mouse model of LAM. Furthermore, therapeutic opportunities of mTORC1 and Wnt inhibitors, singly or in combination, have the potential to advance patient care in TSC/LAM and cancer communities.

The scRNA-seq of pulmonary LAM also enables the development of the connectivity map (CMaP) for drug repositioning and the development for LAM patients [16]. Mahi et al. developed methods for constructing LAM disease transcriptional signatures and CMaP analysis using the scRNA-seq profiling of LAM and control lungs [15] and scRNA-seq data of lung tissue from naïve and sirolimus-treated LAM patients. The new CMaP analysis successfully identified a unique cluster of LAM cells that express LAM marker genes, and differentially and significantly altered the functional enrichment of disease-characterizing signatures in LAM cells. A connectivity analysis also identified sirolimus-treated LAM signature genes. Importantly, mTORC1 inhibitors, including sirolimus, reverted the LAM transcriptional signatures. This methodology development indicates that the precise signature derived from a scRNA-seq data analysis could be the crucial difference between the success or failure to identify effective therapeutic treatments in CMaP analysis. This connectivity analysis also identified additional classes of drugs including Cyclin-dependent kinase (CDK) inhibitors and MEK/extracellular signal-regulated protein kinase (ERK) 1/2 inhibitors that affect the growth and survival of LAM cells.

TSC patients are at risk of developing neurological symptoms, including epilepsy, neuropsychiatric disorders, and autism. In addition to neuron-based pathology, the neuropathological manifestations of TSC include morphological and functional alterations in glial cells [17]. Tang et al. [18] successfully established and characterized a primary murine astrocyte-like cell culture to study the neurologic manifestations of TSC, including the development of cortical tubers, subependymal nodules (SEN), and subependymal giant cell astrocytomas (SEGA) in the brain [18]. The authors developed *Tsc1*-deficient neural cells derived from spontaneously immortalized mouse astrocytes. These primary cells exhibited mTORC1 hyperactivation, transition from astrocytes to neural stem/progenitor cells, and responded to rapamycin treatment in vitro. Tsc1-deficient primary astrocytes developed into SEGA-like lesions, and rapamycin treatment reduced the tumor burden in vivo. This novel SEGA model is a valuable tool for future mechanistic and therapeutic studies.

mTOR signaling controls the survival of brain cells [19], and the dysregulation of mTOR contributes to neurotoxicity. For example, mTOR modulates the neurotoxic effects of a highly addictive psychostimulant methamphetamine (METH), the continuous use of which causes memory loss and cognitive decline. METH causes neuronal cell death by promoting endoplasmic reticulum (ER) stress via the PI3K/Akt/mTOR pathway. In their recent article, Lee et al. [20] reported that a bioactive flavonoid aromadendrin, isolated from *Pinus sibirica*, *Afzelia bella*, and *Chioanathus retusus*, exhibits protective effects against neurotoxicity induced by METH. Using the SH-SY5y neuroblastoma cell line, the authors found that pre-treatment with aromadendrin decreases METH-induced ER stress and attenuates METH-induced autophagy and apoptosis. Mechanistically, aromadendrin acted by restoring mTOR phosphorylation and preserving PI3K/Akt/mTOR signaling [20]. Although the mechanism by which aromadendrin regulates phosphorylation of mTOR remains to be established, this study provides in vitro evidence of the potential attractiveness of aromadendrin as a protector against METH-induced neurotoxicity.

Accumulating evidence links neurological autism spectrum disorders (ASD) with autoimmunity and immune dysfunction [21]. Interestingly, both ASD and autoimmune disorders (AID) demonstrate genetic heterogeneity and heritability and are independently associated with the aberrations in mTOR signaling. Trifonova et al. [22] performed an analysis of several publicly available datasets to assess the potential genetic similarity between ASD and AID. Supporting a key role of the mTOR network in AID, the authors found that up to 67% of AID genes are directly or indirectly related to the mTOR signaling pathway. Intriguingly, the authors’ analysis also led them to hypothesize that some subphenotypes of ASD and AID could comprise a connected set of disorders sharing mTOR signaling as a common aberrant pathway [22]. This research not only reinforces previous observations [21], but also suggests the existence of the immune subtype of ASD as a specific type of autoimmune disorder with an early onset of predominantly behavioral symptoms, which is linked to the dysregulation of the mTOR signaling network. 

The abnormal activation of mTOR is closely related to the pathogenesis of systemic lupus erythematosus (SLE), a common autoimmune disease characterized by the production of autoantibodies against nuclear antigens and multi-organ tissue damage most commonly occurring in joints, kidneys, skin, and blood vessels [23]. Studies from Perl group demonstrated that mTOR is up-regulated in human SLE T cells, leading to the loss of T cell receptor /CD3ζ chain (TCRζ) through HRES-1/Rab-4-dependent lysosomal degradation [24]. Furthermore, SLE Treg cells had upregulation of both, mTORC1 and mTORC2, in IL-21-dependent manner, and the blockage of mTOR in both complexes by prolonged rapamycin treatment normalized Treg function [25]. Importantly, recently completed phase 1/2 clinical trial demonstrated benefits of prolonged (12 months) rapamycin (sirolimus) treatment for patients with clinically active SLE resistance or intolerance to conventional medications [26], warranting follow-up placebo-controlled clinical trials to further evaluate mTOR inhibitors as a therapeutic option for SLE patients. 

Another type of immune system disorder is acquired immunodeficiency syndrome (AIDS), a chronic, potentially life-threatening condition caused by human immunodeficiency virus (HIV). HIV infection and AIDS are associated with an increased risk of cancers, including aggressive lymphomas of B-cell origin, which remain a cause of AIDS-related malignant deaths, despite the widespread use of antiretroviral therapy. mTOR signaling plays a key role in orchestrating cellular responses to viral infections. The susceptibility of CD4 T cells to HIV-1 infection is controlled by mTOR and could be suppressed by catalytic mTOR inhibitors [27], highlighting an important role of mTOR signaling in the early stages of disease. A recent study from Akbay et al. [28] has focused on the molecular mechanisms of the HIV-1-dependent oncogenic transformation of B cells. The authors discovered that HIV-1 Tat activates Akt/mTORC1 signaling, leading to the inhibition of the activation-induced cytidine deaminase (AICDA) transcriptional repressors *c-Myb* and *E2F8* and consequent AICDA activation. Furthermore, the authors demonstrated that the activation of Akt/mTORC1 depends on the DNA damage induced by ROS [28], suggesting the potential role of Ros-Akt/mTORC1-AICDA in increased genomic instability and proliferation, potential contributors to B cell malignancies.

Over the past decade, studies have revealed the importance of the mTOR signaling network in cardiovascular health and diseases, including myocardial infarction, atherosclerosis, and pulmonary arterial hypertension (PAH). PAH is a progressive and rapidly fatal disease defined as a mean pulmonary artery (PA) pressure greater than 20 mm Hg at rest [29]. Key pathophysiological features of PAH include sustained vasoconstriction and remodeling of small PAs due to, at least in part, the aberrant proliferation, survival, and motility of resident pulmonary vascular cells. The state-of-the-art review by Babicheva, Makino, and Yuan [30] summarizes the role, mechanisms of regulation, and function of mTOR signaling in the development of PAH with a specific focus on pulmonary vascular remodeling and sustained vasoconstriction. The article also uncovers the crosstalk between mTOR and other signaling cascades involved in PAH pathogenesis and provides an in-depth discussion of the therapeutic potential of targeting mTOR signaling in PAH.

In addition to its important role in pulmonary vasculature, mTOR regulates cardiac physiology, and multiple molecular mechanisms of mTOR action in cardiomyocytes and cardiac fibroblasts have been identified. It was demonstrated that the up-regulation of mTOR promotes collagen synthesis and proliferation of cardiac fibroblasts, contributing to the development of myocardial fibrosis [31]. Furthermore, pharmacological inhibition of mTORC1 has been proposed as a potentially attractive therapeutic option that offers cardioprotection [31]. Further expanding our understanding of the mechanisms driving cardiac fibroblast proliferation, the study performed by Sharifi-Sanjani et al. [32] uncovered the role of the transcriptional co-activator Yes-associated protein (Yap), the downstream effector of HIPPO cassette and an upstream regulator of the Akt/mTOR signaling pathway [33], in the proliferation of cardiac fibroblasts in the setting of heart failure (HF). Comparison of human left ventricle (LV) tissues revealed that LVs of HF patients have an over-accumulation of Yap, which was accompanied by a down-regulation of HIPPO at the level of large tumor suppressor 1 (LATS1), an elevated phosphorylation of Akt and ERK1/2, and an enrichment of the fibrosis marker connective-tissue growth factor. Similar to these observations, combined in vitro exposure of human ventricular cardiac fibroblasts to two established profibrotic factors, TGFβ and hypoxia, led to up-regulation of Yap, inactivation of HIPPO-LATS1, and increased Akt and ERK1/2 phosphorylation. These mechanistic changes were accompanied by increases in cell proliferation and collagen I production, suggesting that Yap plays a role in cardiac fibroblast proliferation and LV fibrosis. Using this in vitro model, the authors further demonstrated that Yap acts via Akt and in parallel to ERK1/2 and mTORC1 to promote cardiac fibroblast proliferation [32], suggesting the potential attractiveness of targeting Yap/Akt to reduce LV fibrosis in HF.

In conclusion, mTOR signaling plays an important role in normal physiology and human disease and continues to be a topic of active investigation as an attractive target pathway for therapeutic intervention. Although significant progress has been made in uncovering the complexity of mTOR action in various conditions, more studies are underway to further elucidate the mechanisms of mTOR regulation and function and to develop safer and more effective mTOR-targeting agents for therapeutic interventions.

## Data Availability

This editorial article does not include original research data.
